# Generation of imidazolinone herbicide resistant trait in *Arabidopsis*

**DOI:** 10.1371/journal.pone.0233503

**Published:** 2020-05-22

**Authors:** Huirong Dong, Delin Wang, Zhijing Bai, Yuge Yuan, Wei Yang, Yuexia Zhang, Hanwen Ni, Linjian Jiang

**Affiliations:** 1 Key Lab of Pest Monitoring and Green Management, MOA, Department of Plant Pathology, College of Plant Protection, China Agricultural University, Beijing, China; 2 State Key Lab Plant Physiology & Biochemistry, College of Agronomy and Biotechnology, China Agricultural University, Beijing, China; University of Helsinki, FINLAND

## Abstract

Recently-emerged base editing technologies could create single base mutations at precise genomic positions without generation DNA double strand breaks. Herbicide resistant mutations have been successfully introduced to different plant species, including *Arabidopsis*, watermelon, wheat, potato and tomato via C to T (or G to A on the complementary strand) base editors (CBE) at the P197 position of endogenous acetolactate synthase (ALS) genes. Additionally, G to A conversion to another conserved amino acid S653 on ALS gene could confer tolerance to imidazolinone herbicides. However, no such mutation was successfully generated via CBE, likely due to the target C base is outside of the classic base editing window. Since CBE driven by egg cell (EC) specific promoter would re-edit the wild type alleles in egg cells and early embryos, we hypothesized the diversity of base editing outcomes could be largely increased at later generations to allow selection of desired herbicide resistant mutants. To test this hypothesis, we aimed to introduce C to T conversion to the complement strand of S653 codon at ALS gene, hosting a C at the 10^th^ position within the 20-nt spacer sequence outside of the classic base editing window. While we did not detect base-edited T1 plants, efficient and diverse base edits emerged at later generations. Herbicide resistant mutants with different editing outcomes were recovered when T3 and T4 seeds were subject to herbicide selection. As expected, most herbicide resistant plants contained S653N mutation as a result of G_10_ to A_10_. Our results showed that CBE could create imidazolinone herbicide resistant trait in *Arabidopsis* and be potentially applied to crops to facilitate weed control.

## Introduction

CRISPR/Cas9 could efficiently induce DNA double strand breaks (DSB), followed by either non-homology end joining (NHEJ) or homology directed repair (HDR) in plant cells [1]. In plant and mammalian cells, the predominating NHEJ pathway efficiently yielded insertion/deletion (indels) mutations that largely resulted in loss-of-function outcomes [2]. Although HDR could generate desired mutations especially gain-of-function substitutional mutations, it suffered low efficiency [3].

A novel C to T base editor (CBE) was recently developed by fusing cytosine deaminase to nCas9 (D10A), achieving precise and efficient base editing of C bases at 4-8^th^ position in the spacer sequence [4]. Analysis of known herbicide resistant mutations in plants showed that the herbicide target acetolactate synthase (ALS), also known as acetohydroxy acid synthase (AHAS), harbors two potential herbicide resistant mutations as a result of C to T (or G to A on the complementary strand) conversion [5]. One is P197 codon with Cs within the base editing window, and the other is S653 codon with a G that is complimentary to a C outside of the classic base-editing window. Indeed, conversion of C to T at P197 codon was readily achieved in model plant *Arabidopsis* [6], and in many other crops such as watermelon [7], wheat [8] and tomato [9]. The S653 codon of wheat ALS gene was also subject to CBE, however, resulting in only G654 and G655 edits [8]. Currently, the S653N mutation as a result of C to T transition on the complimentary strand was not achieved via CBE.

Mutations at both positions have great agronomic importance. Mutations at P197 confers high level of resistance to sulfonylurea (SU) herbicides in Dupont’s STS soybean varieties [10], while mutations at S653N confers high level of resistance to imidazolinone (IMI) herbicides in many wheat and rice varieties marketed as Clearfield traits [11]. It should be noted that mutations at P197 do not resist IMI herbicides [12,13] and mutations at S653 do not resist SU herbicides [14]. Moreover, simultaneous mutations at both P197 and S653 provided resistance to both SU and IMI herbicides [15]. Therefore, base editing of S653 would introduce resistance to additional ALS-inhibiting herbicides into various plant species.

In this study, we aimed to generate mutations to the codon of S653 in *Arabidopsis* using the CBE tool developed in our laboratory, and detect the resistance level and cross-resistance after S653 mutation. The base editor was driven by a synthetic egg cell (EC) specific promoter to perform base editing in egg cells [6]. The base editing efficiency was quite low at T1 generation; however, re-editing occurred at high efficiency during the reproduction stage of unedited T1 plants producing a vast number of base edited T2 plants [6]. Therefore, we hypothesized that this mechanism can increase the diversity of base editing outcomes, allowing selection of herbicide resistant mutations at S653.

Our results showed diverse base editing outcomes emerged at T2 generation. When T3 and T4 seedlings were selected with imazapic, survived plants harbored a variety of mutations, however, mostly S653N. Our study provided a strategy for developing herbicide resistant crops using current available CBE systems.

## Materials and methods

### Vector construction

The plasmid CRISPR/Cas9 vector used in this study was developed by Wang et al. [16], available at Addgene.com (plasmid number 91707). After digested by *BsaI*, the spacer sequence (5’ GTGCCACCATTTGGGATCAT 3’) was constructed into this plasmid, in which sgRNA was driven by U_6-26_ promoter, and then transformed into *E*. *coli* DH5α. The construct was extracted from *E*. *coli* and then transformed into *Agrobacterium tumefaciens* GV3101 for transformation of *Arabidopsis*. The spacer sequence was designed to target the codon of S653 on ALS gene (GenBank accession number AT3G48560).

### *Arabidopsis* transformation

*Arabidopsis thaliana* Colombia was used for transformation by floral dip method [17]. *Agrobacterium* harboring the binary vector and inoculated into liquid YEP medium supplemented with gentamicin and kanamycin and cultured at 28°C, 200 r min^-1^. *Agrobacterium* was collected at 3000 r min^-1^ for 20 min and resuspended to OD_600_ 0.6–0.8 using 5% sucrose solution supplemented with 0.02% Silwet L-77. The inflorescences of *Arabidopsis* were dipped into this bacterial solution for 0.5–1 min, kept in dark for 24 h, and then moved back to normal growth conditions.

### Transgenic screening

Harvested seeds were surface sterilized with 75% alcohol and 1.2% NaClO solution and placed to MS medium containing hygromycin at 25 mg L^-1^. After incubated in growth chamber (22 ℃, 16 h light, 8 h dark) for 10 d, transgenic plants were picked out and transferred into pots to allow normal growth.

### DNA extraction, PCR and sequencing

When the plants were at the 5-leaf stage, genomic DNA was extracted from about 100 mg leaf tissue via the cetyl trimethylammonium bromide (CTAB) method. Target loci were amplified with DNA polymerase (Takara Bio Inc., Shiga, Japan) and specific primers. The primers primer-F (5’ CTGTTGCTAACCCTGATGCG 3’) and primer-R (5’ AAGCAGGCAGATCAACAACT 3’) were used to amplify the ALS gene fragment. The amplicons were sent to Qingke for Sanger sequencing.

### Herbicide resistance screening

*Arabidopsis* seeds were placed to MS medium supplemented with 0.24 mg L^-1^ imazapic to select resistant mutants. The wild type and S653N mutants acquired from TAIR were used as negative and positive controls. Herbicide resistant mutants were then transplanted into pots. When the plants were at the 5-leaf stage, the DNA was extracted for genotyping.

## Results

### Efficient and diverse base edits achieved at T2 generation when CBE was driven by EC promoter

The spacer sequence targeting S653 was cloned into pHEE901 vector flanked with sgRNA scaffold as previously described [6]. *Arabidopsis* plants were transformed by floral dip method and transgenic plants were selected on MS medium supplemented with hygromycin at 25 mg L^-1^. Fifteen T1 plants were examined and Sanger sequencing showed that no base editing events, in agreement with the previously observed low base editing efficiency at T1 generation [6]. The top four most-prolific lines in seed production among these 15 T1 lines were chosen to examine whether re-editing occurred at T2 generation. In total of 50 T2 seedlings were randomly selected to determinate the base editing outcomes. There are seven Gs in the spacer sequence that could be potentially edited into As, and were distinguished by their positions towards PAM as followed 5’ATG_18_ATCCCG_12_AG_10_TG_8_G_7_TG_5_G_4_CAC3’.

Sanger sequencing data in Table 1 showed that all four lines examined produced base-edited T2 plants with different editing efficiencies varying from 14.3% (line 4#) to 66.7% (line 1#). Moreover, different lines produced different base-editing outcomes, indicating that re-editing could increase diversity of base-editing results. Different from the base-editing results in human cells and other plants [4, 18], our study did not find C to G mutation or indels. In addition, G bases at different positions likely to have different editing efficiencies. For instance, G_5_ and G_7_ were edited at highest frequency and homozygous G to A conversions also occurred at the G_5_ position. Although G_4_ located within base-editing window, no edits occurred at this position. The targeted G_10_ base within the codon of S653 was neither edited. Surprisingly, G to A transitions were detected at G_12_, which is even further away from the base-editing window, in three plants. This result suggested that G_10_ should locate within the reach of CBE, and base editing could happen at this position if more T2 plants were examined.

**Table 1:** Diverse base editing events generated at T2 generation using EC promoter.

T1 lines	T2 genotype	Editing ratio
**1#**	CCGATGATCCCGAGTGGTGGCAC	2/6
	CCGATGATCCCRAGTGRTRGCAC	1/6
	CCGATGATCCCGAGTGGTRGCAC	3/6
**2#**	CCGATGATCCCGAGTGGTGGCAC	5/12
	CCGATGATCCCGAGTGGTRGCAC	3/12
	CCGATGATCCCGAGTGRTGGCAC	1/12
	CCGATGATCCCGAGTGRTRGCAC	2/12
	CCGATGATCCCGAGTGRTAGCAC	1/12
**3#**	CCGATGATCCCGAGTGGTGGCAC	15/18
	CCGATGATCCCGAGTRRTGGCAC	1/18
	CCGATGATCCCGAGTGRTRGCAC	1/18
	CCGATGATCCCRAGTRGTGGCAC	1/18
**4#**	CCGATGATCCCGAGTGGTGGCAC	12/14
	CCGATGATCCCGAGTGGTRGCAC	1/14
	CCGATGATCCCGAGTGRTRGCAC	1/14

R in red stands for the heterozygosity of base A and G. A in red is homozygous edits. 5’CCG3’ in blue is PAM.

**Fig 1:**
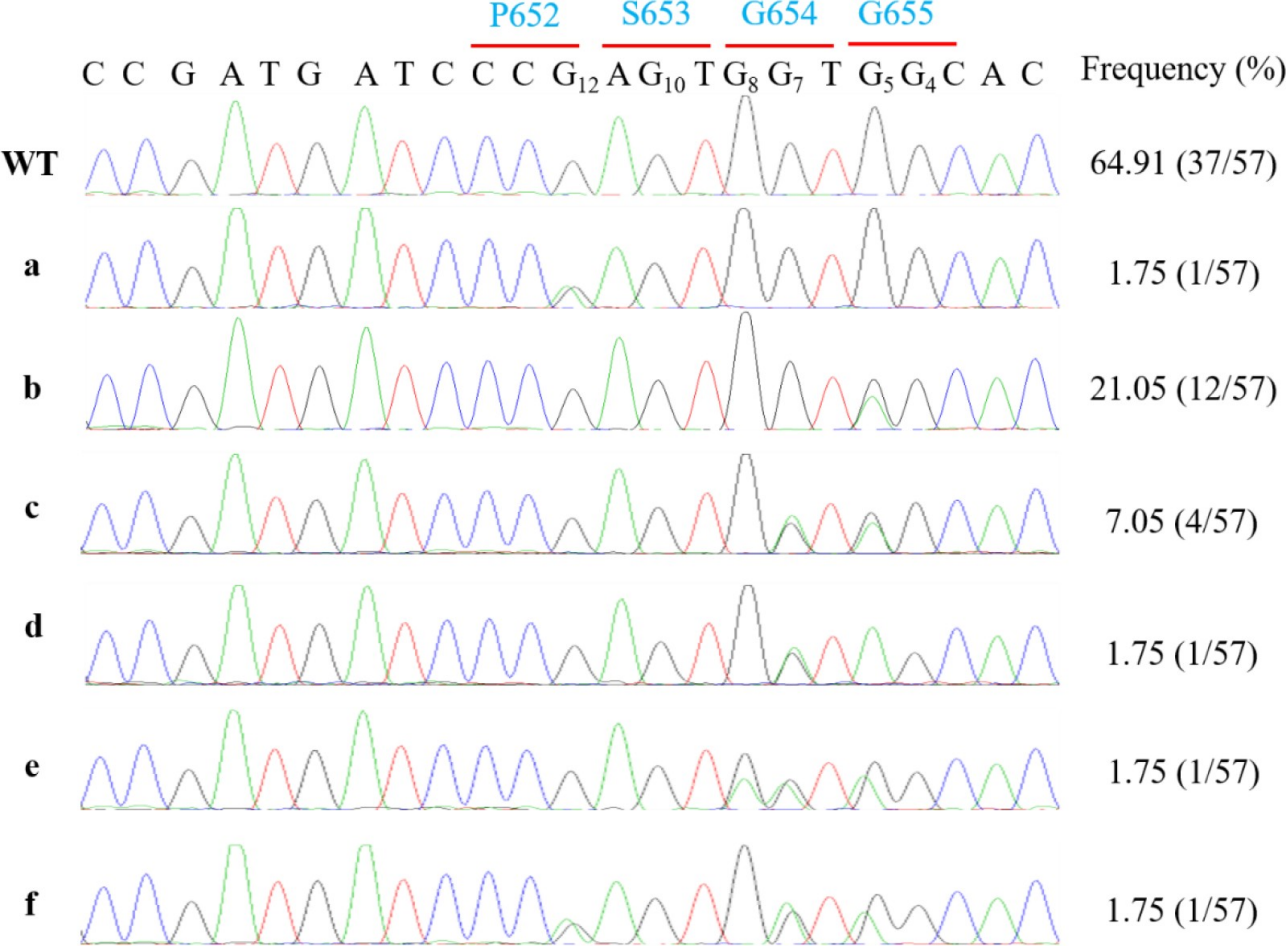
Mutation detection in additional three lines with edits at P652 codon at T2 generation. **a** Heterozygous edit at G12. **b** Heterozygous edit at G5. **c** Heterozygous edits at G7 and G5. **d** Heterozygous edit at G7 and homozygous edit at G5. **e** Heterozygous edits at G5, G7 and G8. **f** Heterozygous edits at G5, G7 and G12. Codons are indicated in blue.

Therefore, additional 57 T2 seedlings in G_12_ edited lines were sampled for Sanger sequencing of PCR amplicons. The results showed that the overall base editing efficiency was 35% (20/57) with similar editing outcomes (Fig 1). Although still no edits occurred at G_10_ position, surprisingly, one plant harbored an edit only at G_12_ position with no edits in the editing window (Fig 1A). This result indicated non-canonical base editing event could emerge as increase of sampled population. Therefore, T3 seeds were harvested for further experiment.

### Editing continued at T3 generation

Eight T2 lines with known genotypes were selected to examine 1) whether the base edited alleles at T2 generation could be inherited to next generation and 2) whether re-editing of wild type alleles could continue.

Sanger sequencing of T3 plants showed that most T2 lines produced offsprings with genotypes that could be explained by Mendel’s law (Table 2). However, some lines likely produced novel mutations at T3 generation when compared with T2 generation. For instance, line 901E-1-6 had heterozygous mutation, i.e., an edited allele and a wild type allele, at G_12_ position at T2 generation, while some of its offsprings had mutations at either G_5_ or G_7_ position. Similar results occurred to lines 901E-1-8 and 901E-13-16. These results did not obey Mendel’s law and were likely due to re-editing of the wild type alleles. Moreover, for lines 901E-1-7 and 901E-13-22, even though genotypes of T3 plants were the same with the parental T2 plants, the absence of wild type T3 plants could also be attributed to re-editing activity.

**Table 2:** The genotype of T3 generation was influenced by re-editing of CBE and Mendel inheritance.

Lines	Genotype of	Genotype of	Genotype
	T2 generation	T3 generation	frequency
**901E-1-6**	CCRAGTGGTGGC	CCRAGTGGTGGC	6/12
		CCAAGTGGTGGC	1/12
		CCGAGTGRTGGC	1/12
		CCGAGTGGTRGC	1/12
		CCRAGTGGTRGC	2/12
**901E-1-7**	CCGAGTGGTRGC	CCGAGTGGTRGC	2/9
		CCGAGTGGTRGC	7/9
**901E-1-8**	CCGAGTGGTRGC	CCGAGTGGTRGC	8/10
		CCGAGTGGTRAC	1/10
		CCGAGTRATRGC	1/10
**901E-13-22**	CCGAGTGGTRGC	CCGAGTGGTRGC	5/8
		CCGAGTGGTAGC	3/8
**901E-13-16**	CCGAGTGGTRGC	CCGAGTGGTRGC	3/8
		CCGAGTGGTAGC	3/8
		CCGAGTGRTGGC	2/8
**901E-1-3**	CCGAGTGRTRGC	CCGAGTGRTGGC	1/7
		CCGAGTGGTRGC	1/7
		CCGAGTGGTAGC	1/7
		CCGAGTGATRGC	1/7
		CCGAGTGRTRGC	3/7
**901E-1-4**	CCGAGTGRTRGC	CCGAGTGRTRGC	8/9
		CCGAGTGATAGC	1/9
**901E-1-16**	CCRAGTGRTRGC	CCRAGTGRTRGC	6/9
		CCAAGTGATRGC	1/9
		CCRAGTGATRGC	1/9
		CCAAGTGRTRGC	1/9

R in red stands for the heterozygosity of base A and G. A in red is homozygous edits.

Moreover, new editing events kept emerging as a result of re-editing. For instance, although no edits at G_4_ position were observed at T2 generation, one T3 plant have homozygous G to A conversions at this position. However, still no edits occurred at G_10_ position in all examined samples.

### G_10_ edits that results in S653N emerged upon herbicide selection

Seeds of 25 T2 lines, each had about 300 seeds, were selected on MS medium supplemented with imazapic herbicide at 0.24 mg L^-1^. Six herbicide resistant plants survived herbicide treatment. Sanger sequencing showed that five had heterozygous S653N mutations and the remaining one harbored homozygous G654D mutation (Table 3).

**Table 3:** The herbicide resistant mutants recovered at T3 generation.

Lines	Genotype	Amino acid change
**901E-13-10-1**	CCRARTGGTGGC	P652P/S653N
**901E-13-10-2**	CCGARTGGTGGC	S653N
**901E013-10-3**	CCGAGTGATGGC	G654D
**901E-13-10-4**	CCRARTGGTGGC	P652P/S653N
**901E-13-8-1**	CCRARTGGTGGC	P652P/S653N
**901E-13-6-1**	CCGARTGGTGGC	S653N

R in red stands for the heterozygosity of base A and G. A in red is homozygous edits.

In order to generate more herbicide resistant mutants, T4 seeds harvested from 12 T3 lines were pooled each together (about 2000 seeds in each line) and selected on MS medium supplemented with imazapic. In total, 76 resistant seedlings survived, and DNA was extracted for genotyping at the 5-leaf stage after transplanted into pots.

**Fig 2:**
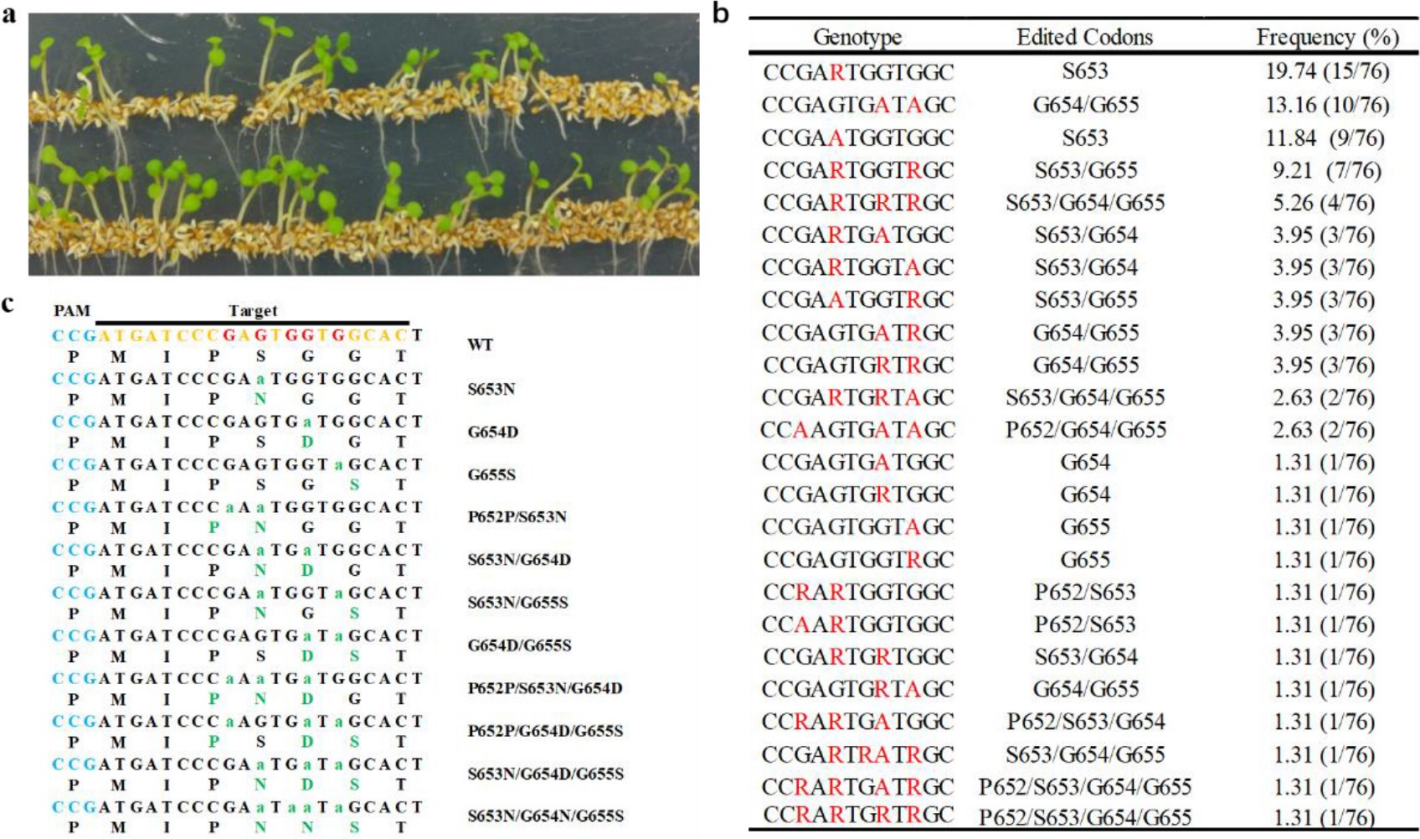
Successful recovery of base edited imidazolinone resistant mutants. **a** Imidazolinone resistant mutants emerged with pooled T4 seeds were subject to MS medium with imazapic at 0.24 mg/l. **b** Genotypes of imidazolinone resistant mutants by Sanger sequencing of PCR amplicons. R in red stands for the heterozygosity of base A and G. A in red is homozygous edits. **c** Imidazolinone resistant alleles revealed by PCR amplicon cloning. Letters in green are modified nucleotides or amino acids.

Sanger sequencing showed that base editing at G_10_ became predominating events in these herbicide resistant plants, i.e., 53 out of 76 plants had homozygous or heterozygous edits at G_10_ resulting in S653N mutation (Fig 2B). Interestingly, 24 plants only have edits at G_10_, nine of which were homozygous mutations. Two plants had simultaneous edits on both P652 and S653, however, the edits on P652 were synonymous edits. Moreover, the rest 27 S653N containing plants also had additional missense edits at G654 or G655. It should be noted that there were 23 herbicide resistant plants without S653N mutation. Nineteen out of these 23 plants seemed to have simultaneous mutations at both G654 and G655 codons and the remaining four had single mutations at either G654 or G655 codon. PCR product cloning sequencing was performed to separate individual alleles, and showed that herbicide resistant plants contained the following missense mutations: S653N, G654D, G655S, S653N/G654D, S653N/G655S, G654D/G655S, P652P/S653N/G654D, P652P/G654D/G655S, S653N/G654D/G655S, S653N/G654N/G655S (Fig 2C).

## Discussion

Although CBE technologies provided efficient C to T conversions, however, challenges remain to be addressed. Firstly, the editing target is restricted by both base editing window and the adjacent PAM sequence. Secondly, base editing efficiency could be largely affected by sequence context, such as the C base in GC context had very low editing efficiency by BE3 [4].

Many Cas9 variants were developed to improve targeting specificity and broaden the targeting freedom. For instance, one SpCas9 variant with expanded PAM was xCas9, and it could recognize a broad range of PAM sequences including NG, GAA and GAT [18]. However, it suffered low efficiency in plants [19]. Alternative variant SpCas9-NG can recognize the NG PAM and significantly increase the targeting flexibility in mammalian cells [20], which soon be proved functional in rice and *Arabidopsis* [21–25]. Some other Cas9 variants have been developed. For example, SaCas9 (NNGRRT), which has been used as a base editing tool in rice [26]. But the other Cas9 variants with different PAMs, such as SpCas9-VQR (NGA) and SpCas9-VRER (NGCG) [27] could also be upgraded to CBEs to expand the targetable sites. Additionally, the SpCas9 based CBE system is also limited by their GC-rich PAM sequences, therefore, the CRISPR-Cas12a (Cpf1) based base editor was developed to perform base editing around TA-rich PAM sequences [28].

Cytosine deaminase is also a key factor in determining the target window as well as preference of sequence context. CBE system was developed using rat APOBEC1 cytidine deaminase linked to nCas9-D10A with base editing window of 5-nt wide [4], however, the base editing efficiency in GC context was very low. The nCas9-D10A fused with activation-induced cytidine deaminase of sea lamprey and achieved specific point mutation of C to T within the target range of five bases [29]. This system has been used successfully in plants, such as potato [30], rice and tomato [31]. CBE with human APOBEC3A was created [32] that performed base editing at a 17nt-wide window in wheat, rice, and potato independent of sequence context. Recently, Thuronyi et al. evolved a new CBE that is 26-fold more efficient than wild type deaminase without sequence context restrains [33]. With these rapid progresses, the limitations of CBE on plants will also be largely addressed.

Zhang et al. tried to create imidazolinone resistant wheat germplasm using CBE targeting to S630 of ALS gene [8], which corresponds to S653 in *Arabidopsis*. As the S630 codon also locates outside of the canonical base editing window, edits mostly occurred at G631 and G632 in the base editing window. Although base edits were observed outside of the canonical base editing window, no edits on S630 were created in wheat [8]. Similarly, in earlier generations, our genotyping efforts also failed to recover S653 edits. However, when selected with imazapic herbicide, S653N emerged and dominated the herbicide resistant mutants in this study, suggesting that S653 might be a more favorable position to confer resistance to imidazolinone herbicides. Indeed, most natural imidazolinone herbicide resistant weeds also carried S653N mutation [34]. Our result suggested combining egg cell specific base-editing with herbicide selection could help to generate low-frequency base editing events, a strategy that might be applied to crops that produced large amount of seeds such as canola.

## Conclusion

In this study, we showed that egg cell specific base editing in *Arabidopsis* continued to produce diverse base-editing events at the following generations. When subject to herbicide selection, rare herbicide resistant edits emerged. This strategy might be potentially applied to crops that produce large amount of seeds, such as canola, for selecting herbicide resistant mutations.
